# RhoKinase (ROCK) Inhibition as a Therapeutic Strategy for Pseudophakic Bullous Keratopathy: A Comprehensive Review

**DOI:** 10.3390/jcm14176093

**Published:** 2025-08-28

**Authors:** Anđela Jukić, Josip Pavan, Biljana Đapic Ivančić, Miro Kalauz, Rajka Kasalica Žužul, Tomislav Jukić

**Affiliations:** 1 Department of Ophthalmology, University Hospital Dubrava, 10000 Zagreb, Croatia; 2 Department of Neurology, University Hospital Centre Zagreb, 10000 Zagreb, Croatia; 3 Department of Ophthalmology, University Hospital Centre Zagreb, 10000 Zagreb, Croatia; 4 School of Medicine, University of Zagreb, 10000 Zagreb, Croatia

**Keywords:** pseudophakic bullous keratopathy, Rho-kinase inhibitors, corneal endothelium, corneal oedema, ripasudil, netarsudil

## Abstract

Pseudophakic bullous keratopathy (PBK) is a vision-threatening corneal complication following cataract surgery, characterised by progressive endothelial cell loss, persistent corneal oedema, and painful epithelial bullae, leading to impaired vision. Corneal transplantation, either penetrating or endothelial keratoplasty, remains the primary treatment but faces challenges such as donor tissue shortages, graft rejection, and limited graft longevity. Recently, Rho-kinase (ROCK) inhibitors have emerged as promising pharmacological alternatives. These agents enhance corneal endothelial cell proliferation, migration, and adhesion, suppress apoptosis, and promote corneal deturgescence and wound healing. Several preclinical and clinical studies have demonstrated the efficacy of ROCK inhibitors in improving corneal clarity, endothelial function, and visual acuity in PBK. Their use has been associated with reductions in corneal oedema, improved endothelial cell density, and delayed or prevented the need for corneal transplantation. A systematic literature search of PubMed, Scopus, and Web of Science databases was conducted, restricted to peer-reviewed English-language articles, ensuring comprehensive coverage. ROCK inhibitors represent a novel pharmacological strategy for PBK prevention and management, potentially reducing dependency on donor grafts. Further research is needed to determine long-term safety, optimal dosing, and efficacy.

## 1. Introduction

Pseudophakic bullous keratopathy (PBK) is a significant complication following cataract surgery, characterised by chronic stromal corneal oedema and painful epithelial bullae due to irreversible loss and dysfunction of corneal endothelial cells [1]. PBK typically manifests between 8 months and 7 years postoperatively and affects approximately 1–2% of cataract surgery cases [1]. Healthy endothelial cells maintain corneal transparency by actively pumping fluid out; however, when endothelial cell density falls below the critical threshold of approximately 500–800 cells/mm^2^, fluid accumulation occurs, resulting in corneal swelling, visual impairment, and discomfort [1]. Clinically, patients experience persistent corneal oedema, decreased visual acuity, glare, and ocular pain caused by ruptured epithelial bullae on the corneal surface. The severity of corneal oedema is classified into grades ranging from mild localised stromal oedema (grade 1) to diffuse bullous keratopathy with severe stromal thickening and folds (grade 4).

Several risk factors predispose patients to PBK, including advanced age, pre-existing endothelial dystrophies such as Fuchs’ endothelial corneal dystrophy (FECD), previous ocular surgeries, shallow anterior chambers, and complicated or prolonged cataract surgeries [1]. The definitive treatment for advanced PBK has traditionally been corneal transplantation, including penetrating keratoplasty (PK) or more modern endothelial keratoplasty procedures like Descemet’s membrane endothelial keratoplasty (DMEK) and Descemet’s stripping automated endothelial keratoplasty (DSAEK). However, corneal transplantation faces considerable limitations, such as donor tissue shortage, technical complexity, and potential complications, including graft detachment, immune rejection, or graft failure over time. Due to these issues, PBK remains one of the leading indications for corneal transplantation globally, second only to FECD [2].

For milder PBK cases or those awaiting transplantation, symptomatic relief through conservative management options such as hypertonic saline (5% sodium chloride) drops or ointments may be utilised. These agents temporarily dehydrate the cornea, offering symptomatic improvement. Bandage contact lenses also serve to protect the corneal surface and relieve discomfort caused by epithelial bullae. Other palliative interventions such as anterior stromal puncture, phototherapeutic keratectomy (PTK), or amniotic membrane transplantation can alleviate refractory pain associated with chronic corneal oedema. However, these treatments do not resolve the underlying endothelial deficiency and typically provide limited visual recovery.

Recently, pharmacological approaches to corneal endothelial dysfunction have gained significant attention, especially following the introduction of Rho-associated protein kinase (ROCK) inhibitors. ROCK inhibitors are serine/threonine kinases involved in cell shape regulation, contractility, adhesion, and apoptosis [3]. In corneal endothelial cells, excessive activation of Rho/ROCK pathways contributes to cellular stress and cell death, while ROCK inhibition facilitates endothelial cell survival, proliferation, and migration, thereby improving fluid management and corneal clarity [4].

Human corneal endothelial cells (CECs) form a monolayer critical for maintaining corneal transparency via barrier and pump functions, but adult human CECs are essentially non-proliferative in vivo. They remain arrested in the G1 phase of the cell cycle due to contact inhibition and high expression of cell-cycle inhibitors [5]. This quiescent state means that endothelial cell loss is normally compensated by cell enlargement and migration rather than mitosis. Rho-associated kinase (ROCK) signaling helps regulate this non-proliferative phenotype through its effects on the cytoskeleton and cell-cycle. Pharmacological ROCK inhibitors (such as Y-27632 and ripasudil) can overcome these inhibitory mechanisms and stimulate CEC proliferation through upregulation of cyclin D and removal of p27 leading to G1/S progression [5]. Consistent with this, ROCK-inhibited CECs exhibit increased proliferation markers (e.g., Ki67 in vivo) and enhanced wound healing capacity [6]. ROCK inhibitors also confer cytoprotective effects: excessive ROCK activity can trigger apoptosis under stress, so its inhibition improves CEC survival and prevents apoptosis [7]. Furthermore, by relaxing actomyosin contractility, ROCK inhibitors induce profound cytoskeletal remodeling—which leads to enhanced endothelial cell spreading and migration helping normally quiescent endothelial cells to repopulate wound areas [6].

Early investigations with the ROCK inhibitor Y-27632 demonstrated its potential to promote corneal endothelial cell regeneration in laboratory studies and animal experiments [4]. Subsequently, ROCK inhibitors such as ripasudil and netarsudil have become available clinically, initially approved for glaucoma treatment by improving aqueous outflow through trabecular meshwork relaxation. These agents additionally exhibit corneal endothelial cell benefits, encouraging surviving endothelial cells to migrate, spread, and cover defects effectively, thus improving corneal oedema [8].

Initial clinical studies involving FECD showed promising outcomes, prompting further exploration of ROCK inhibitors for PBK treatment. Kinoshita et al. notably pioneered a combined approach using intracameral injections of cultured human corneal endothelial cells supplemented with the ROCK inhibitor Y-27632, successfully restoring corneal transparency without the need for traditional donor grafts [9]. Parallel research has indicated that topical ROCK inhibitors alone, such as ripasudil or netarsudil, effectively reduce corneal oedema and enhance visual acuity in milder PBK cases, potentially delaying or negating the need for surgical interventions [1,10].

Given the promising preclinical and clinical results, this review aims to synthesise current evidence on the use of ROCK inhibitors in managing PBK. It will address their mechanisms of action, safety profiles, clinical efficacy and practical implications for clinical ophthalmology practice.

## 2. Methodology

The aim of this narrative review was to evaluate the application of ROCK inhibitors in the treatment of pseudophakic bullous keratopathy (PBK). A comprehensive literature search was performed using three electronic databases: PubMed, Web of Science, and Google Scholar. The search strategy involved keywords such as “Rho-kinase inhibitor,” “ROCK inhibitor,” “pseudophakic bullous keratopathy,” “corneal endothelium,” “corneal edema,” “Y-27632,” “ripasudil,” and “netarsudil,” individually and in various combinations. Medical Subject Headings (MeSH) were applied where appropriate, and Boolean operators such as “AND” and “OR” were utilised to refine searches and ensure the inclusion of highly relevant studies.

All peer-reviewed studies published in English up to July 2025 were considered eligible for inclusion, provided they specifically addressed the pharmacological or regenerative use of ROCK inhibitors for PBK. Both preclinical studies (including animal models, laboratory-based experiments, and ex vivo investigations using human corneal tissues) and clinical research (encompassing case reports, case series, cohort analyses, and clinical trials) were included if relevant outcomes were reported. Studies were excluded if they were non-English, non-peer-reviewed, lacked original data, focused exclusively on surgical interventions without pharmacological use of ROCK inhibitors, or addressed unrelated conditions such as glaucoma.

## 3. Results

The findings were systematically summarised into preclinical evidence, clinical evidence from cell-based regenerative therapies, clinical evidence from topical pharmacological therapies for prevention and treatment of PBK.

### 3.1. Preclinical Evidence of ROCK Inhibition on Corneal Endothelium

Initial evidence supporting the efficacy of Rho-kinase (ROCK) inhibitors in corneal endothelial healing originated from laboratory and animal studies. Okumura et al. (2013) [8] demonstrated that ROCK inhibition significantly promotes endothelial cell regeneration. In a rabbit model with induced central endothelial injury, topical administration of the ROCK inhibitor Y-27632 markedly accelerated endothelial wound closure and restored corneal transparency compared to untreated controls [8]. The treated corneas displayed a higher density of regenerated endothelial cells, re-established pump function, and reduced corneal oedema, whereas untreated corneas remained persistently oedematous [8].

These findings were subsequently confirmed using ripasudil, a clinically available ROCK inhibitor. In a later study, Okumura et al. (2016) [4] evaluated ripasudil 0.4% eye drops in a rabbit model following endothelial scraping injury. In vitro exposure to ripasudil notably enhanced human corneal endothelial cell (HCEC) proliferation. In vivo application resulted in significantly improved corneal endothelial wound healing, with full transparency restoration achieved in five of six treated rabbit eyes within two weeks. All untreated control eyes remained severely oedematous [4].

These preclinical results demonstrated that ROCK inhibition promotes endothelial cell migration, adhesion, and potentially stimulates residual or transplanted endothelial cells to proliferate, a process rarely observed under physiological conditions in adult human corneas.

### 3.2. ROCK Inhibitors to Prevent PBK and Corneal Endothelial Damage

In addition to facilitating corneal endothelial wound healing, evidence suggests that ROCK inhibitors exert protective effects during surgical stress.

In an ex vivo human corneal study, Okumura et al. found that activating the RhoA/ROCK pathway induced endothelial cell apoptosis, whereas ROCK inhibition protected cells from apoptosis [8]. This observation provides a rationale for the prophylactic use of ROCK inhibitors during procedures known to induce endothelial trauma, such as cataract surgery.

Multiple clinical studies have evaluated topical Rho kinase (ROCK) inhibitors as a strategy to protect the corneal endothelium during or after intraocular surgery.

In patients at high risk (e.g., Fuchs endothelial dystrophy, low endothelial cell density), prophylactic ROCK inhibitor eye drops have shown significant benefit. A recent prospective randomized trial in late-stage Fuchs dystrophy found that ripasudil 0.4% given 1 month preoperatively through 2 months post-cataract surgery preserved corneal clarity and thickness compared to controls. At 3 months, eyes treated with ripasudil had stable central corneal thickness (CCT) and improved visual acuity, whereas control eyes had a significant CCT increase and no visual gain; endothelial cell density (ECD) remained stable in both groups [11].

Similarly, a randomized controlled trial in Fuchs patients by Keeratidamkerngsakul et al. reported that 1 month of postoperative ripasudil drops led to ~0% endothelial cell loss centrally vs. ~7% loss in controls (paracentral ECD loss 0.4% vs. 7.3%). Treated eyes also maintained lower corneal light scatter: corneal densitometry remained unchanged with ripasudil but worsened in controls. Although central corneal thickness increased slightly in both groups over 3 months, ripasudil-treated eyes showed overall better endothelial function and only mild transient hyperemia as a side effect [12].

Fujimoto et al. (2021) [13] conducted a controlled study assessing ripasudil 0.4% administered twice daily immediately following cataract surgery in patients with low preoperative endothelial cell counts. Patients treated with ripasudil exhibited significantly less postoperative corneal oedema in the first postoperative week (+1.25% increase in corneal thickness) compared to controls (+5.97%, *p* = 0.0037). Moreover, endothelial cell loss at three months was significantly lower in ripasudil-treated patients (approximately 4.5%) compared to untreated controls (approximately 14.1%, *p* = 0.0003) [13]. These findings highlight the potential for ROCK inhibitors to preserve endothelial integrity postoperatively, potentially mitigating the risk of subsequent pseudophakic bullous keratopathy.

Further supporting this preventive role, Alkharashi et al. (2024) [14] conducted a prospective comparative trial in cataract surgery patients. In this study, patients receiving ripasudil three times daily for five days postoperatively showed significantly less endothelial cell loss at 12 months (median 4.5%) compared to untreated controls (median 12.8%, *p* = 0.001). Correspondingly, central corneal thickness measurements indicated less persistent corneal oedema in treated patients. Importantly, no adverse effects were reported from short-term prophylactic ripasudil use [14].

Antonini et al. (2022) reported a case series involving three patients with pre-existing endothelial compromise (Fuchs endothelial corneal dystrophy) undergoing cataract surgery, treated with topical ripasudil 0.4% four times daily [15]. In one case that developed PBK postoperatively, topical ripasudil effectively resolved stromal oedema, improved visual acuity, and delayed the necessity for keratoplasty. Two additional patients received ripasudil prophylactically before and after cataract extraction, successfully maintaining clear corneas postoperatively and avoiding the anticipated ~13% rate of endothelial failure in similar high-risk patients. The authors concluded that topical ripasudil could serve as an effective adjuvant therapy to reduce the incidence of postoperative PBK in patients with compromised endothelial cell density (ECD).

These studies indicate that perioperative ROCK inhibition can mitigate surgical endothelial injury and prevent development of PBK, especially in high-risk patients.

### 3.3. ROCK Inhibitor-Augmented Cell Therapy for PBK

A pivotal advancement in PBK management has been the integration of ROCK inhibition with regenerative endothelial cell therapy. This innovative approach involves the intracameral injection of laboratory-expanded human corneal endothelial cells (CECs) supplemented with ROCK inhibitors, enhancing cell adhesion, migration, and functional regeneration on the recipient’s Descemet’s membrane.

The first clinical evaluation of this technique by Kinoshita et al. (2018) involved 11 patients with pseudophakic or aphakic bullous keratopathy who received intracameral injections of approximately 100,000 cultured human CECs combined with the ROCK inhibitor Y-27632 [9]. All patients demonstrated resolution of corneal oedema, restoration of corneal transparency, and substantial visual acuity improvement within two months post-injection. Central corneal thickness normalised to approximately 500–600 µm, and corneal endothelial cell density (ECD) increased to an average of ~1900 cells/mm^2^ (range 947–2833 cells/mm^2^) after six months. Notably, no significant inflammation, rejection, or ocular complications were reported, confirming the safety and feasibility of this therapeutic strategy [9].

Long-term follow-up data further validated the durability of this approach. At two years post-treatment, 10 of the original 11 patients retained clear corneas with central thickness values below 630 µm, indicating a success rate of approximately 91% [2]. A subsequent five-year evaluation confirmed sustained treatment efficacy, with 10 of 11 patients maintaining normal corneal transparency and central ECD averaging 1257 cells/mm^2^. Visual outcomes remained favourable, with best-corrected visual acuity (BCVA) between 20/25 and 20/40 or better in most cases, comparable to outcomes from traditional endothelial keratoplasty techniques such as DMEK or DSAEK. Importantly, no cases of immunologic rejection occurred [16].

Following these encouraging initial results, combined ROCK inhibitor and endothelial cell injection therapy advanced through phase II and phase III clinical trials in Japan, involving a total of 27 patients with PBK [17]. At six months post-procedure, pooled results showed that 94.1% (16 of 17 evaluable eyes) achieved the primary efficacy endpoint of maintaining ECD ≥ 1000 cells/mm^2^. Additionally, 82% demonstrated central corneal thickness < 630 µm without residual oedema, with mean thickness reductions of approximately 187 µm from baseline. Visual acuity consistently improved in all treated patients. Adverse events, observed in 89% of cases, were generally mild and transient, including brief intraocular pressure elevation or mild inflammation, with no serious safety issues reported. These pivotal clinical trials culminated in regulatory approval in Japan (2022) of the combined cultured human CEC and ROCK inhibitor therapy, commercially marketed as nenadendocel (formerly HVJ-E or Aurion cell therapy), marking the first regenerative endothelial cell treatment authorised for corneal endothelial diseases [17].

The recent prospective, double-masked Escalón trial (n = 22) provided additional evidence supporting this approach [18]. In this study, 18 eyes with PBK underwent endothelial polishing followed by intracameral injection of 1 × 10^6^ cultured human CECs suspended in three different concentrations (10–100 µM) of Y-27632. At 12 months, treated eyes showed a significant reduction in mean central corneal thickness from 697 µm to 571 µm (−126 µm), along with marked visual acuity improvements (mean BCVA from 0.995 to 0.330 logMAR). Approximately 89% of eyes gained at least 0.3 logMAR. Importantly, all tested ROCK inhibitor concentrations demonstrated similar efficacy, and no serious adverse events were recorded, confirming treatment safety and efficacy even at lower inhibitor concentrations [18].

Currently licensed for clinical application in Japan, ROCK inhibitor-augmented endothelial cell therapy is also undergoing further evaluation at multiple international centres. Concurrently, early-stage research is exploring induced pluripotent stem cell-derived corneal endothelial substitutes similarly supplemented with ROCK inhibitors, aiming to improve cell adhesion, survival, and functional outcomes [19].

### 3.4. Topical ROCK Inhibitor Therapy in PBK

Several clinical studies have explored the off-label use of topical ROCK inhibitor eye drops for managing PBK.

Tseng and Feder (2023) retrospectively evaluated topical ripasudil therapy (0.4%, four times daily) in five patients with persistent corneal oedema following anterior segment surgery, including three cases with PBK [10]. After 2–4 weeks of treatment, four patients showed marked improvements in corneal transparency and visual acuity. Notably, one PBK patient experienced transient benefits, with corneal oedema recurring and progressing upon cessation of ripasudil, eventually requiring endothelial keratoplasty. Mild conjunctival hyperaemia was the only reported adverse effect, and no significant complications such as infection or neovascularisation were observed.

In the retrospective study from 2024, Lavy et al. assessed ripasudil 0.4% eye drops in 16 patients with chronic corneal oedema unresponsive to standard medical therapies [20]. Among this cohort, approximately 31% had PBK, alongside cases of post-PK oedema, failed DMEK grafts, and other aetiologies. Following an average treatment duration of five months, significant improvements were observed in mean best-corrected visual acuity (BCVA), improving from logMAR 1.106 (~20/250 Snellen) to logMAR 0.56 (~20/70, *p* = 0.0308). Mean central corneal thickness (CCT) also decreased significantly from 619.5 µm to 572.5 µm (*p* = 0.0479). Although a slight increase in endothelial cell counts (from 849 to 875 cells/mm^2^) was noted, this change was not statistically significant.

Erdinest et al. (2025) further detailed topical ripasudil use specifically for PBK in a case series involving three patients who had chronic corneal oedema for 3–6 months and no prior improvement on maximal medical therapy [1]. Patients were treated with ripasudil 0.4% drops three times daily for durations ranging from 3 to 11 months. All patients showed notable clinical improvement. In one case, CCT decreased from 594 µm to 584 µm with visual acuity improving from 0.08 to 0 logMAR (20/20). Another patient exhibited a significant reduction in CCT from 686 µm to 654 µm and improvement in BCVA from 1.0 to 0.39 logMAR (~20/50). The third patient, treated over 11 months, experienced substantial corneal clarity improvement (CCT 582 µm to 540 µm) and visual acuity enhancement (0.3 to 0.04 logMAR, approximately 20/40 to 20/11). All patients reported symptomatic relief and none required subsequent corneal transplantation during follow-up [1].

The largest retrospective cohort study up to date, Erdinest et al. included 96 patients with PBK from various etiologies; post-cataract surgery n = 32, Fuchs endothelial corneal dystrophy n = 29, post-Descemet membrane endothelial keratoplasty n = 25 and post-penetrating keratoplasty n = 10, who were treated with topical ripasudil for 5.2 ± 2.3 months. They reported significant reductions in CCT and visual acuity, particularly in post-cataract surgery and FECD related corneal edema. These data support the role of ripasudil as a non-surgical pharmacotherapy for corneal edema, with potential to delay and in some cases obviate surgical intervention [21].

## 4. Discussion

This review highlights two principal clinical applications of ROCK inhibitors in managing pseudophakic bullous keratopathy (PBK): as adjunctive therapy to regenerative endothelial cell injection and as topical pharmacologic treatment to support endogenous endothelial function.

Both strategies utilise ROCK inhibitors’ unique ability to promote endothelial cell migration, enhance cell survival, reduce apoptosis, and encourage formation of a stable, functional endothelial monolayer [8].

ROCK inhibition has notably transformed regenerative endothelial cell therapies, enabling injected cultured cells to effectively adhere to the recipient’s Descemet’s membrane. Without ROCK inhibitors, injected cells often aggregate or undergo anoikis due to detachment. Kinoshita et al.’s groundbreaking trial demonstrated a >90% success rate in creating a sustainable endothelial monolayer in PBK patients treated with cultured endothelial cells plus ROCK inhibitor Y-27632, achieving outcomes comparable to traditional keratoplasty but without graft implantation [9]. Importantly, these results were sustained over long-term follow-ups, confirming the procedure’s durability [16]. Advantages include minimal invasiveness, lower induced astigmatism, faster visual recovery, and no graft-related immunologic rejection. However, widespread adoption remains constrained by the need for specialised cell-culture facilities, regulatory approvals, and associated costs. Ongoing commercial developments, such as pre-loaded cell-plus-ROCK inhibitor injections, may expand clinical availability.

Emerging evidence suggests that perioperative Rho-kinase (ROCK) inhibition can mitigate surgical endothelial trauma and thereby help prevent progression to pseudophakic bullous keratopathy (PBK) in high-risk eyes. Mechanistic support comes from ex vivo human corneas, where pre-exposure to a ROCK inhibitor significantly reduced endothelial apoptosis after simulated phacoemulsification, reinforcing the rationale for perioperative use [22]. Building on convergent data from systematic reviews and clinical experiences, perioperative topical ROCK inhibitors are associated with less postoperative corneal edema, smaller increases in central corneal thickness, lower light scatter, and a trend toward reduced endothelial cell loss after phacoemulsification in eyes with compromised endothelium, while adverse effects are generally mild and transient (most commonly conjunctival hyperemia) [23].

Topical ROCK inhibitors, primarily ripasudil and netarsudil, offer immediate clinical relevance due to their availability. Evidence increasingly supports their efficacy in mild-to-moderate PBK, particularly in patients retaining some viable endothelial cells. Improvement typically includes visual gains of 2–3 lines and corneal thinning between 30–100 µm [1]. Partial responders may achieve symptom relief, improved visual comfort, and reduced dependence on keratoplasty. Furthermore, preoperative topical ROCK inhibitor therapy could enhance surgical outcomes by reducing pre-surgical corneal oedema and associated complications [20,24]. Severe or advanced cases lacking viable endothelial cells are less likely to benefit, emphasizing the importance of careful patient selection.

ROCK inhibitors have also demonstrated potential when combined with other procedures. Notably, topical ROCK inhibitor therapy showed potential when combined with Descemetorhexis (DSO + ROCK) or post-DSAEK and post-DMEK, to accelerate corneal deturgescence and graft adherence.

Beyond PBK, topical ROCK inhibitors have shown promise in managing other corneal endothelial disorders. Studies in FECD reported significant visual improvement and deturgescence following topical ROCK inhibitor application [25]. Even rare conditions, such as iridocorneal endothelial syndrome (ICE), have responded positively [26]. ROCK inhibitors may enhance endothelial health through modulation of cytoskeletal tension, cell proliferation pathways, Na/K ATPase pump activity, and potentially anti-fibrotic and anti-inflammatory effects [4,27,28].

Regarding safety, topical ROCK inhibitors exhibit a generally favourable profile. Common side effects include mild, transient conjunctival hyperaemia, conjunctival haemorrhages, and reversible cornea verticillata. Rare transient epithelial oedema has been observed in compromised corneas but resolves upon medication discontinuation [2,4]. In regenerative cell injection trials, no significant ROCK-specific toxicities were reported, with mild events attributed to procedural factors rather than the inhibitor itself [17]. Thus, topical ROCK inhibitors present a promising risk–benefit balance for patients.

Future research should aim to establish long-term efficacy, optimal dosing, and durability of treatment response. Randomised controlled trials comparing topical ROCK inhibitors to standard care would clarify their clinical role further. Exploration into the precise biological mechanisms underpinning ROCK inhibitor efficacy will enhance understanding and potentially expand therapeutic indications.

## 5. Conclusions

Pseudophakic bullous keratopathy (PBK) remains a significant challenge following cataract surgery, traditionally necessitating corneal transplantation in advanced stages. The advent of ROCK inhibitors has introduced novel therapeutic strategies aimed at enhancing corneal endothelial cell survival, promoting regeneration, and reducing corneal oedema. Evidence from preclinical and clinical studies highlights two primary approaches: regenerative endothelial cell therapy augmented by ROCK inhibitors, and topical pharmacological treatment to optimise residual endothelial cell function.

Clinical outcomes for both strategies have demonstrated substantial promise, often restoring corneal clarity and improving visual acuity without immediate transplantation. Topical ripasudil or netarsudil offer a practical and effective management option, especially for mild-to-moderate PBK or in patients unsuitable for immediate surgical intervention, potentially delaying or eliminating the need for keratoplasty. ROCK inhibitor-augmented cell therapy represents a transformative shift towards minimally invasive regenerative ophthalmology and is expected to become more accessible with future commercial developments. Current evidence also supports ROCK inhibitors as a noninvasive, topical strategy with a preventive role in reducing endothelial injury and delaying, or potentially averting, PBK after cataract surgery.

Despite encouraging evidence, further research, including randomised controlled trials, are necessary to establish standardised treatment protocols, optimal dosing regimens, patient selection criteria, and long-term outcomes.

This review paper shows that given their favourable safety profile and unique mechanism of action, ROCK inhibitors represent an important advancement in the therapeutic management of PBK and are likely to become integral components of future ophthalmic practice.

## Data Availability

Not applicable.
